# Evaluation of Self-Degradation and Plugging Performance of Temperature-Controlled Degradable Polymer Temporary Plugging Agent

**DOI:** 10.3390/polym15183732

**Published:** 2023-09-11

**Authors:** Hualei Xu, Liangjun Zhang, Jie Wang, Houshun Jiang

**Affiliations:** 1 Cooperative Innovation Center of Unconventional Oil and Gas, Yangtze University, Wuhan 430100, China; 2 Hubei Key Laboratory of Drilling and Production Engineering for Oil and Gas, Yangtze University, Wuhan 430100, China; 3 State Key Laboratory of Petroleum Resources and Prospecting, China University of Petroleum, Beijing 102249, China

**Keywords:** temporary plugging diversion fracturing, plugging performance, temporary plugging agent, braking agent, degradation performance

## Abstract

Temporary plugging diversion fracturing (TPDF) technology has been widely used in various oil fields for repeated reconstruction of high-water-cut old oil wells and horizontal well reservoir reconstruction. Previous studies have carried out in-depth study on the pressure-bearing law and placement morphology of different types of temporary plugging agents (TPAs) in fractures, but there are relatively few studies on TPA accumulation body permeability. To solve this problem, an experimental device for evaluating the TPA performance with adjustable fracture pores is proposed in this paper. Based on the test of fracturing fluid breaking time and residue content, the low damage of fracturing fluid to the reservoir is determined. The TPA degradation performance test determines whether the TPA causes damage to the hydraulic fracture after the temporary plugging fracturing. Finally, by testing the TPA pressure-bearing capacity and the temporary plugging aggregation body permeability, the plugging performance and the aggregation body permeability are determined. The results show the following: (1) Guar gum fracturing fluid shows good gel-breaking performance under the action of breaking agent, and the recommended concentration of breaking agent is 300 ppm. At 90~120 °C, the degradation rate of the three types of TPAs can reach more than 65%, and it can be effectively carried into the wellbore during the fracturing fluid flowback stage to achieve the effect of removing the TPA in the fracture. (2) The results of the pressure-bearing performance of the TPA show that the two kinds of TPAs can quickly achieve the plugging effect after plugging start: the effect of ZD-2 (poly lactic-co-glycolic acid (PLGA)) particle-and-powder combined TPA on forming an effective temporary plugging accumulation body in fractures is better than that of ZD-1 (PLGA) pure powder. There are large pores between the particles, and the fracturing fluid can still flow through the pores, so the ZD-3 (a mixture of lactide and PLGA) granular temporary plugging agent cannot form an effective plugging. (3) The law of length of the temporary plugging accumulation body shows that the ZD-2 combined TPA has stronger plugging ability for medium-aperture simulated fracture pores, while the ZD-1 powder TPA has stronger plugging ability for small aperture simulated fracture pores, and the ZD-3 granular TPA should be avoided alone as far as possible. This study further enriches and improves the understanding of the mechanism of temporary plugging diverting fracturing fluid.

## 1. Introduction

**Figure 1:**
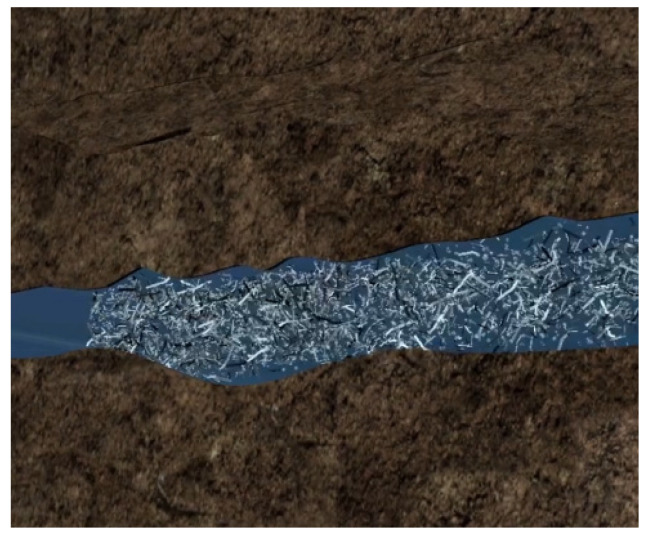
TPA enters the main fracture.

**Figure 2:**
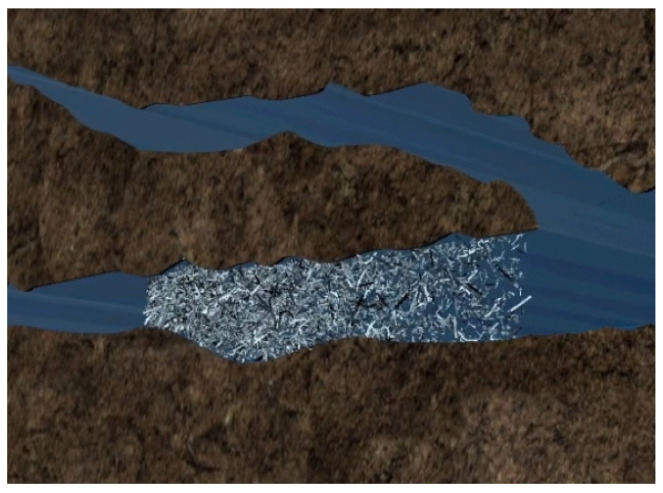
TPA plugs the main fracture, forcing the subsequent injection of fracturing fluid to open new fractures.

For the development of unconventional reservoirs with low porosity and permeability, it is often necessary to use hydraulic fracturing to reconstruct the reservoir, but conventional hydraulic fracturing technology is easy to form a single symmetrical fracture in the reservoir; the reservoir reserves cannot be used effectively and the effect of fracturing is limited [1,2]. With the further development of petroleum exploration and development, temporary plugging diversion fracturing (TPDF) technology has become the main technology for low-permeability reservoir reconstruction [2,3,4]. TPDF has been widely used in various oil fields to carry out repeated reconstruction of high-water-cut old oil wells and horizontal well reservoir reconstruction. This is an effective development technology for low-permeability oil fields and complex formations containing unconventional oil and gas resources [3,4,5]. Temporary plugging technology pumps TPAs into the target layer under certain conditions, uses the difference of permeability in the layer to plug the high permeability layer, lets the injected fluid flow to the low permeability layer and achieves the purpose of changing the flow direction of the original liquid and reconstructing the low-permeability layer (as shown in Figure 1 and Figure 2). TPAs are a kind of treatment agent widely used in oil field production, which can plug high-permeability layers, force fracturing fluid to change direction and realize fracturing reconstruction in the remaining oil accumulation area. It has been used in field operation [4,5], and temporary plugging fracturing technology can play the role and has the potential of fracturing in reservoir development to the maximum [6,7]. At present, various types of TPAs have been widely used in oil fields. A large number of performance evaluation experiments of TPAs have been carried out indoors, but due to technical reasons, there are still some problems such as unclear mechanism of TPAs, imperfect evaluation methods of temporary plugging effects, unclear TPA pressure-bearing laws and aggregation morphology [7,8,9].

The plugging ability of TPAs is affected by many factors such as TPA formulation (concentration, particle size, ratio and shape), fracture morphology and fracture opening [10,11,12,13]. Because the fracture model often cannot directly reflect the influence of the real fracture on the plugging effect, it is impossible to accurately adjust the fracture opening to explore the plugging ability of TPAs [14,15,16]. In addition, the use of TPAs has different degrees of formation damage [17]. The traditional triaxial temporary plugging fracturing physical simulation experiment device has a thin pipeline and cannot pump the TPA. At the same time, due to the limitation of wellbore structure, the borehole barefoot interval is not long enough, so it is difficult for the TPA to enter the fracture, and it is impossible to carry out TPDF simulation [18]. In the case of ensuring the smooth injection, strong plugging pressure-bearing performance is a necessary condition for the TPA. At present, there are many studies on fiber temporary plugging at home and abroad, but few studies on particles, combined particles or particle/fiber combination [19,20]. The combined diversion material has higher plugging efficiency and pressure-bearing performance for fractures [21,22,23,24]. At present, there is a lack of related research on the optimization of the combination process of its materials, and the amount of addition in the field construction is mostly based on experience. The excellent properties of the TPA determine the success of the diversion fracturing process in the fracture [25,26,27]. Therefore, on the basis of clarifying the plugging mechanism of temporary plugging materials, this paper carries out the experiment of plugging pressure-bearing performance of TPAs in fractures and optimizes the type and combination of TPA, so as to better guide the field construction.

In order to solve the problem that it is impossible to determine the plugging, pressure-bearing performance and applicable fracture pore size of various TPAs based on experiments, an experimental device for evaluating the performance of TPAs with adjustable fracture pore size is proposed in this paper, and the effect of temporary plugging is evaluated by monitoring and analyzing the pump injection pressure curve. Meanwhile, according to the experimental phenomena and results, the plugging and pressure-bearing laws of different types of TPAs on different fracture pore sizes during the plugging process can be explored. By observing the morphology of the temporary plugging aggregation body, the migration and accumulation laws of TPAs can be inferred. The device has the characteristics of low cost, low energy consumption, flexible module and simple operation, which can provide a solid experimental basis for the study of TPA performance. In addition, it can provide experimental reference and theoretical basis for reservoir reconstruction and deep fluid flow diversion in the process of oil and gas field development, and then serve oil and gas exploitation to improve oil recovery and increase production and efficiency.

## 2. Methodology

### 2.1. Fluid and Additives Property

The guar gum fracturing fluid used in this paper was TPDF, 0.5 wt% hydroxypropyl guar gum (JK101) + 1 wt% stabilizer (KWD-105H, cationic polyacrylamide) + 0.75 wt% regulator (YC-150, Polyether surfactants) + 0.5 wt% crosslinking agent (YP-150, organoboron). The water used for liquid preparation was deionized water, and the breaking agent was ammonium persulfate. All were provided by Beijing Kemaishi Oilfield Chemical Technology Co., Ltd. (Beijing, China). The TPAs consisted of several types of large particles, medium particles, small particles and powder. Their main component is degradable preformed particle materials (DPPM); ZD-1 and ZD-2 are composed of poly lactic-co-glycolic acid (PLGA); ZD-3 is composed of lactide and PLGA (50:50). The material objects and characteristics are shown in Table 1.

### 2.2. Experimental Device and Procedure

In order to reflect the easy degradation, low residue and effective plugging properties of the TPDF fluid system, the following experimental test types were designed: (1) fracturing fluid gel breaking time and residue content test; (2) degradation performance test of TPA; (3) pressure-bearing capacity test of TPA; (4) temporary plugging aggregation body permeability test. The following will first introduce the relevant experimental steps, and then introduce the temporary plugging pressure experimental device and the adjustable fracture pore width temporary plugging aggregation body experimental device, as well as the relevant experimental steps.

### 2.3. Fracturing Fluid Gel-Breaking Time and Residue Content Test

According to the standard (SY/T 5107-2016: The evaluation measurement for properties of water based fracturing fluid) of fracturing fluid gel-breaking time test [28,29], the water bath temperature of the device was 80 °C. The fracturing fluid system containing 200 ppm, 300 ppm, 400 ppm and 500 ppm of breaking agent concentration was prepared, and the solution was heated in a water bath pot to test the viscosity of the gel-breaking fracturing fluid at different time points. When the viscosity of the fracturing fluid reaches 2.0 mPa·s, or is obviously close to water, it is considered that the fracturing fluid is completely broken. The residue content of gel-breaking fracturing fluid was tested by vacuum filtration.

### 2.4. Degradation Performance Test of TPA

In order to ensure that the TPA is completely degraded and has no damage to the formation after the temporary plugging fracturing, it is necessary to test whether the TPA has good degradation performance. The main steps include the following: (1) Prepare several parts of 2 wt% KCl simulated formation water, weigh the TPA ZD-1, ZD-2 and ZD-3 with a mass of 5 g, respectively, and pour the three TPAs into a glass container containing 100 mL simulated formation water to seal. (2) Put the glass container containing the TPA into a constant temperature water bath heated to 90 °C, remove the container at different time periods, observe and weigh the remaining weight of the TPA and calculate the degradation rate of the TPA (as shown in Equation (1)) [30]. (3) Adjust the temperature of the water bath to 100 °C, 110 °C and 120 °C, and repeat steps 1~2 to test the degradation rate of the temporary plugging agent at different temperatures.

(1) $$\gamma =\frac{m-∆}{m}\times 100\%$$

where *γ* is degradation rate of TPA, %; *m* is the initial mass of the TPA, g; ∆ is the remaining mass after the degradation of the TPA, g.

### 2.5. Temporary Plugging Pressure Test Device and Procedure

In the process of temporary plugging fracturing, the TPA is transported to the main fracture by guar gum fracturing fluid, forming an accumulation body in the fracture, which plays a role in plugging the main fracture, opening the fracture in other directions and finally forming a complex fracture network structure [31,32]. Therefore, the accumulation body formed by the TPA must have a certain pressure-bearing capacity to achieve the plugging effect. In order to simulate the real fracturing fractures in the formation, the rock fractures after fracturing were perfectly reproduced by using high-strength materials through 3D scanning printing technology, and the 3D-printed simulated fractures with repeatable experiments were obtained. The pressure-bearing capacity test experiments of different types of TPAs were carried out indoors.

**Figure 3:**
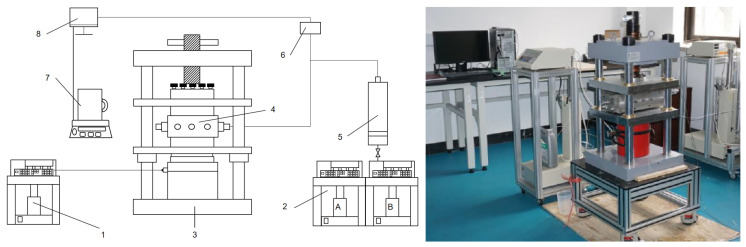
Testing device for TPA aggregation body formation and pressure-bearing capacity [33].

**Figure 4:**
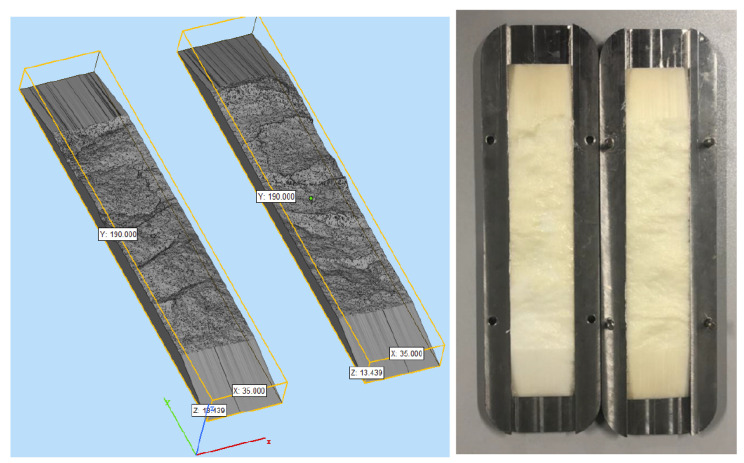
3D-printed rock plate physical diagram. Note: the size of the 3D-printed rock plate was L 190.0 mm × W 35.0 mm × H 13.439 mm.

The schematic diagram of the experimental device shown in Figure 3, including (1) confining pressure pump; (2) constant speed and constant pressure injection pump (A and B); (3) hydraulic fixing device; (4) the diversion chamber with 3D rock plate; (5) an intermediate container for placing fracturing fluid; (6) pressure-monitoring device; (7) liquid-metering device at the outlet end; (8) data acquisition system. The 3D-printed rock plate is shown in Figure 4.

The experimental steps of TPA aggregation body formation and pressure-bearing capacity test are as follows: (1) prepare temporary plugging fracturing fluid; (2) install 3D fracture simulation; (3) install steel blocks on the upper and lower sides of the 3D-printed simulated fracture, put the diversion chamber into the press and add confining pressure on both sides; (4) pour the temporary plugging fracturing fluid into the intermediate container and connect the pipeline equipment; (5) set the injection pump flow rate to 60 mL/min, and inject the temporary plugging fracturing fluid into the fracture simulator; (6) when the pressure at the injection end reaches 10 MPa, stop the injection and continue for three mins to observe the pressure drop; (7) remove the injection pressure, stop the experiment and clean the equipment; (8) replace the type or concentration of TPA, and repeat steps 1~7.

### 2.6. Experimental Device and Procedure for Temporarily Plugging Aggregation Body with Adjustable Fracture Pore Width

#### 2.6.1. Design and Construction of Experimental Device

**Figure 5:**
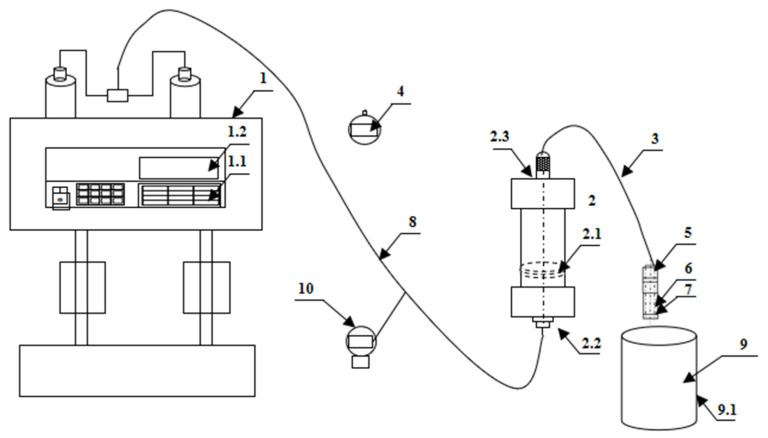
The overall structure diagram of the experimental device. Note: 1—constant speed constant pressure pump; 1.1—system control panel; 1.2—electronic display; 2—intermediate container; 2.1—piston; 2.2—liquid inlet; 2.3—liquid outlet; 3—diversion pipeline; 4—timer; 5—temporary plugging pipeline interface; 6—TPA accumulating pipeline; 7—fracture pore simulation plug; 8—injection line; 9—collection bucket; 10—pressure gauge.

The overall structure schematic diagram of the experimental device for TPA performance evaluation with adjustable fracture pore width is shown in Figure 5. The experimental device consists of a pumping system, a fracturing fluid injection device, a measuring device and a temporary plugging aggregation body forming device. The core part of the experimental device is a temporary plugging aggregation body formation device.

#### 2.6.2. Introduction of Temporary Plugging Aggregation Body Formation Device

**Figure 6:**
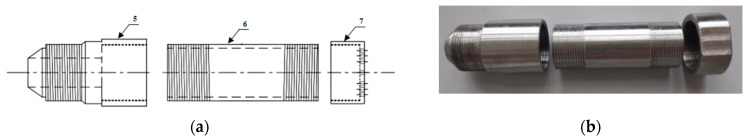
Schematic diagram and physical picture of temporary plugging aggregation body forming device. (**a**) Schematic diagram; (**b**) physical picture. Note: No 5—connector, No 6—Temporary blocking aggregate analog device body, No 7—outlet end.

**Figure 7:**
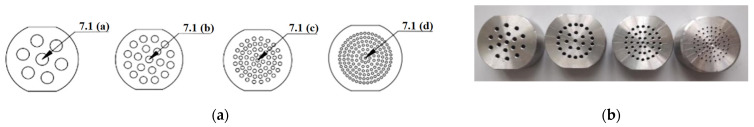
Schematic diagram and physical picture of simulated plugs with different apertures. (**a**) Schematic diagram; (**b**) physical picture. Note: 7.1 (a)—super-large-aperture simulated fracture pores; 7.1 (b)—large-aperture simulated fracture pores; 7.1 (c)—medium-aperture simulated fracture pores; 7.1 (d)—small-aperture simulated fracture pores. The length of TPA-accumulating pipeline is 8.5~9.0 cm, and the inner diameter is 2.5 cm. The fracture pore size of the simulated plug is 0.90 mm for small aperture, 1.38 mm for medium aperture, 2.16 mm for large aperture and 2.50 mm for super-large aperture.

The main parts of the temporary plugging aggregation body forming device are the temporary plugging pipeline interface, the TPA-accumulating pipeline and the fracture pore simulation plug. As shown in Figure 6, the components of the temporary plugging pipeline interface, the TPA-accumulating pipeline and the fracture pore simulation plug are all connected by threads. There are simulated fracture pores at the bottom of the fracture pore simulation plug. The inner diameter of the interface between the diversion pipeline and the temporary plugging pipeline is the same. The connection between the temporary plugging pipeline interface and the diversion pipeline is treated by the leakage prevention process to prevent the fracturing fluid leakage. The inner diameter of the temporary plugging pipeline interface is smaller than that of the TPA-accumulating pipeline, which can reduce the influence of fracturing fluid backflow on the temporary plugging aggregation body morphology and ensure that the temporary plugging aggregation body is formed in the TPA-accumulating pipeline. The inner wall of the TPA-accumulating pipeline is smooth and connected by threads before and after. As shown in Figure 7, the simulated plugs with different apertures are as follows: super-large aperture 7.1 (a), large aperture 7.1 (b), medium aperture 7.1 (c) and small aperture 7.1 (d), from left to right. During the experiment, the simulation plug with different apertures was replaced to simulate the different sizes of formation fracture pores. The temporary plugging performance and pressure-bearing law of the TPA for different sizes of fracture pores were investigated through multiple sets of experiments.

#### 2.6.3. Main Experimental Steps

The main experimental steps of the TPA performance evaluation for the adjustable fracture pores include the following: (1) Connect the experimental equipment in turn according to the experimental device (Figure 6). An intermediate container 2 is filled with an appropriate amount of TPA fracturing fluid required for the experiment. (2) The flow parameters of the constant speed constant pressure pump 1 are set through the system control panel 1.1, and the constant speed constant pressure pump 1 pushes the piston in the intermediate container to inject the fracturing fluid into the diversion pipeline 3 at a fixed flow rate. (3) The fracturing fluid flows through the diversion pipeline 3 to the temporary plugging aggregation body formation device. When fracturing fluid starts to flow out the simulated plug 7, the timer 4 is pressed to start the timing, and the pressure at the corresponding time of the pressure gauge 10 is observed and recorded. When the pressure indication reaches 10 MPa, the constant speed constant pressure pump 1 is closed, and the time pressure curve is drawn according to the recorded data. (4) Replace different TPA fracturing fluid or different aperture fracture simulation plug 7 and, repeat steps 1~3. (5) Take out the temporary plugging accumulation body from the TPA-accumulating pipeline 6, and observe the temporary plugging accumulation body morphology to study the TPA accumulation law and the compaction morphology of the accumulation body. (6) Analyze the relationship curve between time and pressure, and explore the plugging and pressure-bearing performance of different types of TPAs on simulated holes of different apertures.

## 3. Results and Discussion

### 3.1. Fracture Fluid Gel Breaking and Residue Content

**Figure 8:**
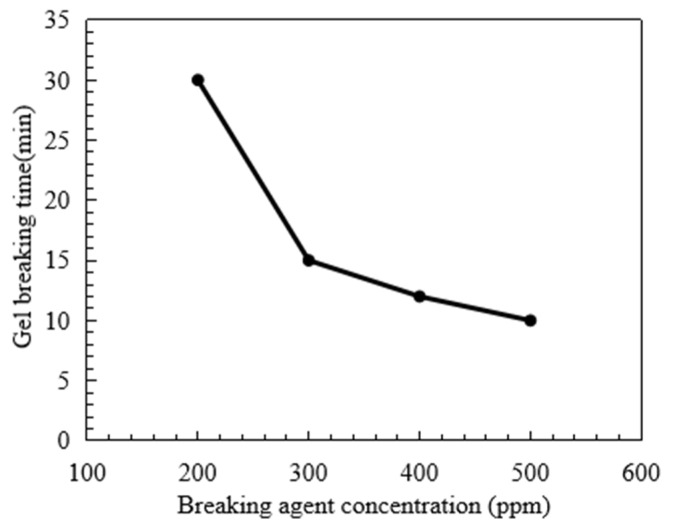
Influence of the breaking agent concentration on the fracturing fluid breaking time.

**Figure 9:**
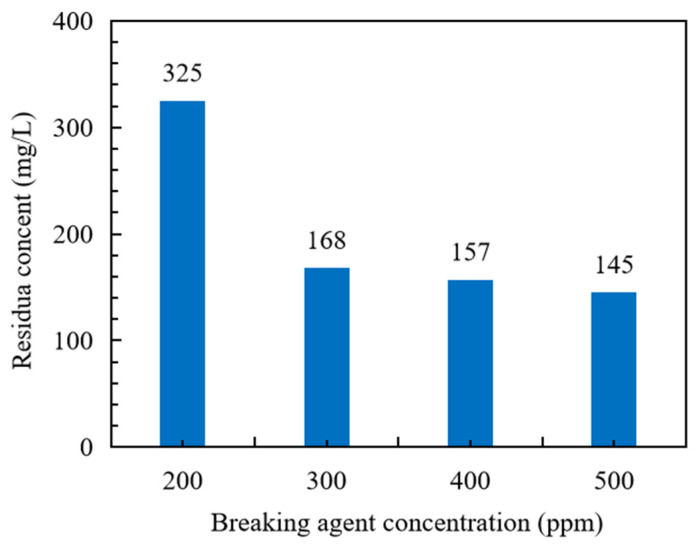
Residue content of fracturing fluid after gel breaking.

The gel-breaking performance of fracturing fluid and residue content are two of the main indexes to measure whether the fracturing fluid is harmful to the reservoir. Figure 8 shows the corresponding gel-breaking time of fracturing fluid when the breaking agent concentration increases. When the breaking agent concentration is 200 ppm, 300 ppm, 400 ppm and 500 ppm, the gel-breaking time of fracturing fluid is 30, 15, 13 and 10 min, respectively. When the breaking agent concentration increases from 200 to 300 ppm, the corresponding gel-breaking time is reduced by about one time. In the process of further increasing the breaking agent concentration, the gel-breaking time of the fracturing fluid is only reduced by a small margin. Figure 9 shows the residue content of fracturing fluid after gel breaking under the action of different breaking agent concentrations. When the breaking agent concentration reaches 300 ppm, the residue content is reduced from 325 to 168 mg/L. Further, the content of the breaking agent increased, and the residue content only decreased slightly. Considering the gel-breaking time and residue content of fracturing fluid, the recommended concentration of breaking agent is 300 ppm.

### 3.2. Degradation Performance of TPA

**Figure 10:**
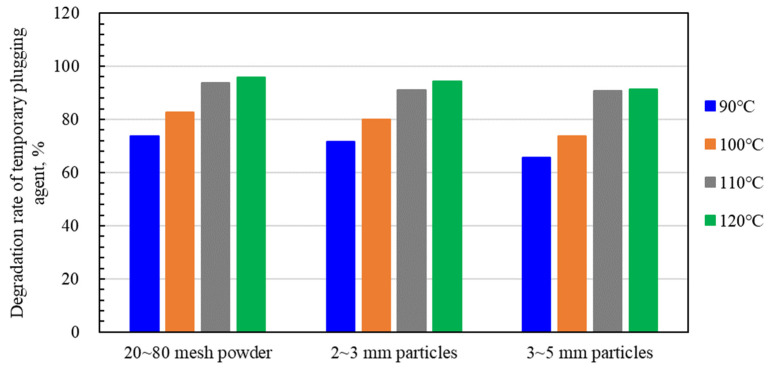
Degradation rate test results of different types of TPA after 96 h.

The degradation performance of a single material composed of three types of TPA was tested, and the results are shown in Figure 10. It can be seen that when the temperature is 90 °C and the water bath time is 96 h, the degradation rate of the three single types of TPAs ranges from 65.5 to 73.6%, the degradation rate of 20~80 mesh powder reaches the highest, 73.6%, and the degradation rate of 6~8 mm particle material reaches the lowest, 65.5%. When the temperature reaches 110 °C, the degradation rates of 20~80 mesh powder, 3~6 mm particles and 6~8 mm particles are 93.6, 91.0 and 90.6%, respectively, all of which are above 90%. When the temperature further increased to 120 °C, the degradation rate of the three types of TPAs did not increase significantly. The degradation performance of TPA in solution is related to temperature and water contact area. The contact area between particle and liquid is much smaller than that of powder and fiber. Therefore, under the same water bath time and temperature, with the particle material size increasing, the corresponding degradation performance becomes weaker. Overall, the degradation rate of the three types of TPAs can reach more than 65% at 90~120 °C and can be effectively carried into the wellbore during the fracturing fluid backflow stage to achieve the effect of removing the TPA in the fracture.

### 3.3. Pressure-Bearing Capacity of TPA

**Figure 11:**
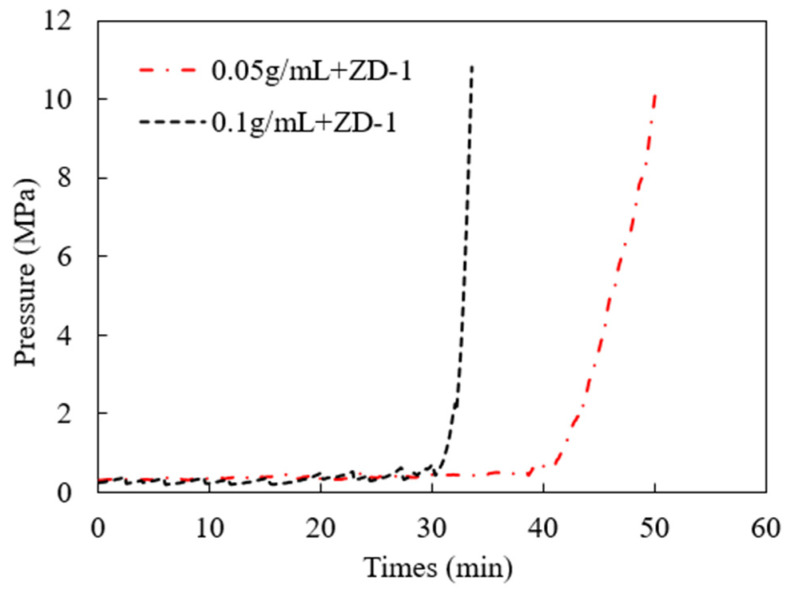
Injection pressure curve of ZD-1 TPA, 10 MPa.

**Figure 12:**
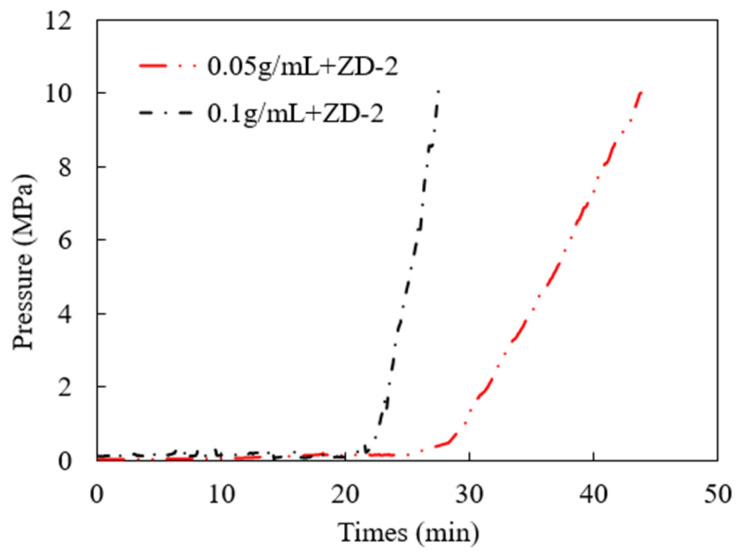
Injection pressure curve of ZD-2 TPA, 10 MPa.

**Figure 13:**
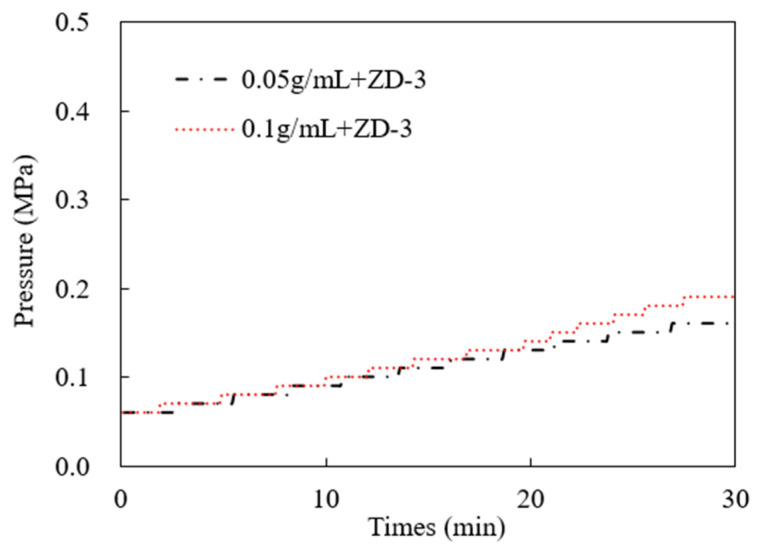
Injection pressure curve of ZD-3 TPA, 10 MPa.

**Figure 14:**
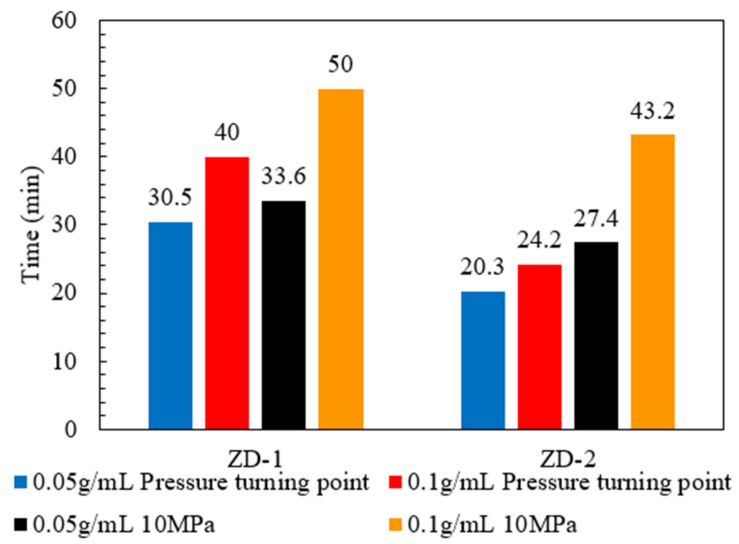
Comparison between the pressure turning point of TPA and the time when the injection pressure reaches 10 MPa.

According to the TPA concentration commonly used in temporary plugging hydraulic fracturing, a temporary plugging fracturing fluid system with a concentration of 0.05 g/mL and 0.1 g/mL was prepared. The fracture width of 6 mm was selected to test the pressure-bearing capacity of the TPA. The results are shown in Figure 11, Figure 12 and Figure 13. Both ZD-1 and ZD-2 can form effective plugging in fractures, and ZD-3 cannot form plugging in fractures. The analysis shows that ZD-1 belongs to the powder TPA, and ZD-2 belongs to the powder-and-particle combined TPA. The two types of TPAs gradually form an effective plugging system in the fracture through the accumulation bridging effect. Because ZD-2 contains particles with larger particle size, it can preferentially accumulate bridges in fractures and lay powder PTAs to quickly achieve the effect of plugging fractures. Taking the TPA with a concentration of 0.05 g/mL as an example (Figure 14), when the pressure turning point is reached, the corresponding time of ZD-1 and ZD-2 is 30.5 and 20.3 min, respectively. When the pressure reaches 10 MPa, the corresponding time of ZD-1 and ZD-2 is 33.6 and 27.4 min, respectively. When the pressure turning point is generated, the difference between ZD-1 and ZD-2 is 10 min, but when it finally reaches 10 MPa, the time difference between the two is 6.2 min, indicating that after the plugging start, the two types of TPAs can quickly achieve the plugging effect. ZD-3 belongs to the granular PTA. Although it can form a rapid plugging effect in the fracture, due to the large pores between the particles, the fracturing fluid can still flow through the pores and cannot form an effective plugging (Figure 13).

### 3.4. Temporary Plugging Aggregation Body Permeability

**Figure 15:**
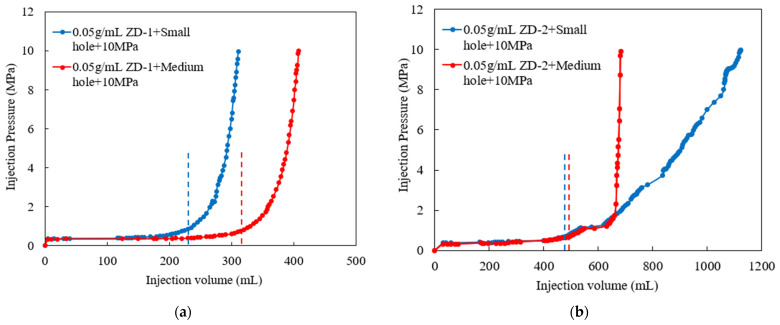
Pressure curves of 0.05 g/mL TPA. (**a**) ZD-1 TPA; (**b**) ZD-2 TPA.

**Figure 16:**
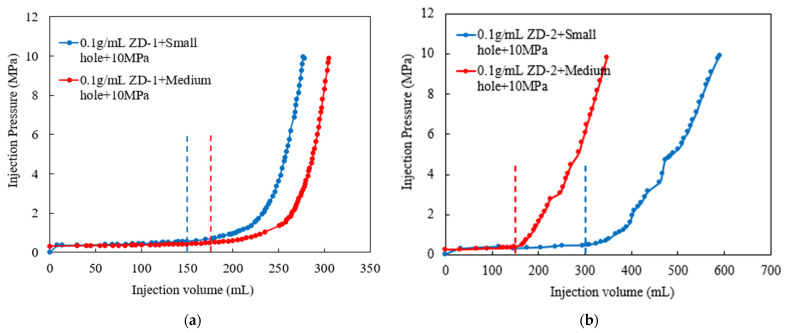
Pressure curves of 0.10 g/mL TPA. (**a**) ZD-1 TPA; (**b**) ZD-2 TPA.

In Section 3.3, the pressure-bearing capacity of the TPA was studied. The ZD-2 particle-and-powder combined TPA has the ability to bridge and plug fractures quickly. It is verified whether the aggregation body formed by the TPA has permeability, which plays a key role in the pressure-bearing capacity of temporary plugging. The following will focus on the permeability characteristics of the temporary plugging aggregation body. According to the injection time and temporary plugging pressure curve drawn by the experimental data, as shown in Figure 15 and Figure 16, the constant flow rate is set to 60 mL/min during the experiment, so the abscissa injection volume represents the product of the corresponding injection time and injection rate.

According to the analysis of experimental results, the formation of pressure-bearing temporary plugging aggregation bodies indicates that the combined TPA and powder TPA have expected plugging performance for simulated fracture pores. In the experiment, the pressure-bearing law of the TPA reflects its plugging performance. The faster the pressure rising is, the stronger the plugging ability of the TPA for the simulated fracture pores is. It can be seen from the time pressure curves (Figure 15 and Figure 16) that ZD-2 combined TPA and ZD-1 powder TPA have different plugging abilities for different apertures. At high concentration, ZD-2 has better plugging ability for medium-aperture simulated fracture pores, while ZD-1 has better plugging ability for small-aperture simulated fracture pores.

The length and permeability of the temporary plugging aggregation body and corresponding pressure data were statistically analyzed in the TPA plugging process with different types of fracture pores. The results are shown in Table 2. The aggregation body permeability of ZD-1 powder TPA is generally smaller than that of particle-and-powder combined TPA, and the injection amount is also smaller than that of ZD-2 when the plugging pressure reaches 10 MPa. This is the reverse of the amount of TPA needed when plugging is formed in the fracture. Compared with the fracture model, the pore model only plugs the pores, mainly to test the aggregation body permeability. Therefore, less leakage in the formation of the plugging process can achieve effective plugging. The law of the length of the temporary plugging aggregation body shows that the ZD-2 combined TPA has a stronger plugging ability for medium-diameter simulated fracture pores, while the ZD-1 powder TPA has a stronger plugging ability for small-diameter simulated fracture pores.

### 3.5. Morphological Analysis of Temporary Plugging Aggregation Body

**Figure 17:**
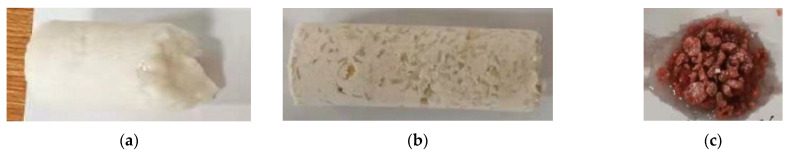
Physical pictures of three types of TPA aggregation body. (**a**) ZD-1 TPA; (**b**) ZD-2 TPA; (**c**) ZD-3 TPA.

The temporary plugging aggregation bodies formed in Section 3.4 were photographed and analyzed, as shown in Figure 17a–c. From the appearance analysis, the aggregation body formed by ZD-1 TPA is short and compact, and the aggregation body of ZD-2 TPA clearly shows the morphology of mutual doping of particles and powders. However, the aggregation body formed by ZD-3 TPA is loose and cannot form effective plugging in fracture or pore models.

**Figure 18:**
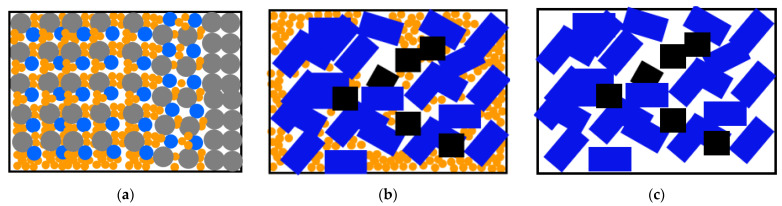
Spatial structure diagram of three types of TPA aggregation body. (**a**) ZD-1 TPA; (**b**) ZD-2 TPA; (**c**) ZD-3 TPA.

By analyzing the morphology characteristics of aggregation bodies formed by three types of TPAs, the spatial structure diagram of TPA aggregation bodies is shown in Figure 18a–c. Figure 18a shows that the 20 mesh powder with larger particle size of powder TPA preferentially forms accumulation and a plugging effect at the pore, and the subsequent injection of 20~80 mesh powder gradually forms accumulation and a plugging effect. As a whole, it presents a compressed entity of large, medium and small particles. Figure 18b shows the effect of the particle material of ZD-2 TPA on bridging and the powder material filling pores, but the overall compaction effect is weaker than that of ZD-1 powder TPA. Figure 18c shows that the pores of the temporary plugging aggregation body formed by ZD-3 granular TPA are large, and fracturing fluid can still flow through the pores between the aggregation body particles.

## 4. Conclusions

In view of the problem that it is impossible to determine the plugging and pressure-bearing performance of various TPAs and applicable fracture pore sizes according to experiments, an experimental device for evaluating the performance of TPAs with adjustable fracture pore size is proposed in this paper. Based on the test of fracturing fluid gel-breaking time and residue content, the low damage of fracturing fluid to the reservoir is determined. The degradation performance test of the TPA determines whether the TPA causes damage to the hydraulic fracture after the temporary plugging fracturing. Finally, by testing the pressure-bearing capacity of the TPA and the permeability of the temporary plugging aggregation body, the plugging performance and the permeability of the aggregation body are determined. The main conclusions and recommendations are as follows:

(1) Guar gum fracturing fluid shows good gel-breaking performance under the action of gel-breaking agent. When the amount of breaking agent is 200~500 ppm, the corresponding gel-breaking time and residue content are 30~10 min and 325~145 ppm, respectively. When the breaking agent concentration increases from 200 ppm to 300ppm, the gel-breaking time and residue content of fracturing fluid increase the most, and the gel-breaking agent concentration is recommended to be 300 ppm. At 90~120 °C, the degradation rate of the three types of TPAs can reach more than 65%. In the fracturing fluid backflow stage, it can be effectively carried into the wellbore to achieve the effect of removing the TPA in the fracture.
(2) The results of the pressure-bearing capacity of the TPA show that the effect of the ZD-2 particle-and-powder mixed TPA on forming an effective aggregation body in the fracture is better than that of the ZD-1 pure powder TPA. When the pressure turning point is generated, the time difference between ZD-1 and ZD-2 is 10 min, but when it finally reaches 10 MPa, the difference between the two is 6.2 min, indicating that after the plugging start, the two types of TPAs can quickly achieve the plugging effect. Due to the large pores between particles, the fracturing fluid can still flow through the pores, and the ZD-3 granular TPA cannot form an effective plugging.
(3) The performance evaluation experimental device of TPAs with adjustable fracture pores was used to carry out indoor evaluation experiments on three types of preferred TPAs. ZD-2 combined TPA and ZD-1 powder TPA successfully formed temporary plugging aggregation bodies with good pressure-bearing performance, while granular TPA failed to form a pressure-bearing temporary plugging aggregation body. The length law of temporary plugging aggregation bodies shows that the ZD-2 combined TPA has stronger plugging ability for medium-aperture simulated fracture pores, while the ZD-1 powder TPA has stronger plugging ability for small-aperture simulated fracture pores. By analyzing the aggregation morphology of the TPA, it can be seen that the ZD-2 temporary plugging aggregation body is relatively loose, while the ZD-1 powder TPA has a relatively compact aggregation morphology, which is the main influence on the formation length and permeability of the temporary plugging aggregation body.

## Figures and Tables

**Table 1:** Characteristics and physical diagram of temporary plugging material.

TPA Type	TPA Characteristic	TPA Physical Diagram
ZD-1 Powder TPA	Mainly powder, almost no particles. It consists of 20 mesh:40 mesh:80 mesh, three kinds of powder according to the volume ratio of 1:1:1.	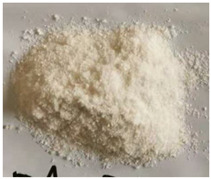
ZD-2 Combined TPA	Powder and particles are evenly combined according to 1:3. Among them, the powder is 40 mesh, and the length of the large particle material is 2~3 mm.	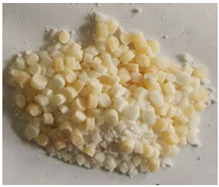
ZD-3 Granular TPA	It is composed of irregular particles. The particle length varies from 3 to 5 mm.	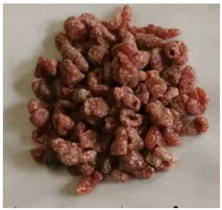

**Table 2:** Summary of key parameters in the formation of temporary plugging aggregation body.

TPA Type	TPA Concentration, g/mL	Aperture Type	The Corresponding Injection Volume and Pressure When Pressure Rising, mL/MPa	Injection Volume (10 MPa), mL	Temporary Plugging Aggregation Body Permeability, mD
ZD-2 Combined TPA	0.10	Small	311/0.489	591	24.30
		Middle	149/0.380	348	24.56
ZD-2 Combined TPA	0.05	Small	450/0.596	1123	23.34
		Middle	465/0.625	682	23.44
ZD-1 Powder TPA	0.10	Small	150/0.565	278	18.90
		Middle	165/0.462	305	18.76
ZD-1 Powder TPA	0.05	Small	230/0.844	315	17.88
		Middle	315/0.753	410	17.92
ZD-3 Granular TPA	Unable to form effective plugging accumulation body (10 MPa)				

## Data Availability

Not applicable.

## Nomenclature

DPPM	Degradable preformed particle materials
ZD-1	Powder type temporary plugging agent
ZD-2	Temporary plugging agent of particle and powder combination
ZD-3	Granular temporary plugging agent
3D	Three dimensional
KCl	Potassium chloride
TPDF	Temporary plugging diversion fracturing
TPA	Temporary plugging agent
